# Targeted congenital cytomegalovirus screening as part of the newborn hearing screening programme in Northeast England

**DOI:** 10.1007/s00431-025-06612-9

**Published:** 2025-11-11

**Authors:** Alexander J. Hagan, Eleri J. Williams, Philip Lindsey, Kate Johnston, Steven Powell, Janet Berrington, Marieke Emonts

**Affiliations:** 1 School of Medicine, Newcastle University, Framlington Place, Newcastle Upon Tyne, NE2 4HH UK; 2 Department of Paediatric Immunology, Infectious Diseases and Allergy, Newcastle Upon Tyne Hospitals NHS Foundation Trust, Great North Children’s Hospital, Newcastle Upon Tyne, NE1 4LP UK; 3 Audiology Department, Newcastle Upon Tyne Hospitals NHS Foundation Trust, Newcastle Upon Tyne, UK; 4 Department of Paediatric Ear, Nose and Throat Services, Newcastle Upon Tyne Hospitals NHS Foundation Trust, Newcastle Upon Tyne, UK; 5 Department of Neonatal Medicine, Newcastle Upon Tyne Hospitals NHS Foundation Trust, Newcastle Upon Tyne, UK; 6 Translational and Clinical Research Institute, Newcastle University, Newcastle Upon Tyne, UK

**Keywords:** Neonatal screening, Congenital cytomegalovirus infection, Sensorineural hearing loss, Audiology, Diagnostic

## Abstract

Neonates with congenital CMV (cCMV) with hearing loss at birth have improved hearing and developmental outcomes when treatment is started within the first 4 weeks of life. This report retrospectively evaluates the performance of a targeted diagnostic pathway for ‘otherwise well’ neonates who fail their newborn hearing screen. Approximately 10% of ‘otherwise well’ neonates who failed their newborn hearing screen and were subsequently diagnosed with sensorineural hearing loss were identified as having cCMV, highlighting the pathway’s diagnostic utility. Our findings do, however, identify a missed subgroup—infants with complex medical needs requiring prolonged neonatal hospital stays—who are often referred for audiology assessment too late for timely cCMV testing. These findings support the need to optimise early referral pathways to ensure that all at-risk neonates are offered diagnostic cCMV testing within the recommended time frame.

**What is Known:**

• *Treatment of symptomatic cCMV infection in neonates started timely before 4 weeks of life, improves hearing and developmental outcomes*.

• *cCMV screening for all neonates is not available in all countries*.

**What is New:**

• *This report demonstrates the diagnostic utility of a regional pathway embedded within the UK Newborn Hearing Screening Programme for identifying ‘otherwise well’ neonates with cCMV-associated hearing loss timely, allowing start of treatment within the first 4 weeks of life*.

• *Alternative strategies are required for premature and unwell hospitalised neonates*.

## Background

Congenital CMV (cCMV) is the most common congenital infection globally, affecting approximately 0.48% of livebirths in high-income countries [1]. While only 10–15% of infants present as symptomatic at birth, all remain at risk of developing permanent sequelae [2]. Among the most significant late effects, cCMV is a leading non-genetic cause of childhood hearing loss in developed countries, accounting for approximately 10–15% of cases [3, 4]. Approximately 7–18% of asymptomatic infants may develop hearing loss throughout early childhood, with variable onset, course and severity, underscoring the importance of early identification to prevent missed treatment opportunities [5, 6].

Recent evidence, including the 2024 European Guidelines [7], suggests that antiviral treatment initiated within the first 4 weeks of life can improve hearing outcomes, with potential benefits also observed when treatment is commenced up to 3 months of age [4, 5]. In the absence of universal cCMV screening, targeted neonatal testing integrated within the UK Newborn Hearing Screening Programme (NHSP) offers a feasible alternative [8]. In the North of Tyne region of the North East of England any child who has ‘no clear response’ in one or both ears on their newborn hearing screen is referred for a diagnostic audiological assessment, with the intent of completing this within the first 3 weeks of life. Infants whose first audiological tests are suggestive of sensorineural hearing loss (SNHL) or auditory neuropathy spectrum disorder are urgently referred for a urine CMV PCR.

While cCMV testing in infants with unexplained SNHL is an established clinical practice in some tertiary centres, there is a lack of data highlighting the potential value of a systematically integrated, region-wide pathway that is embedded within the UK NHSP. The aim of the current project is to assess the diagnostic utility of our integrated pathway in identifying ‘otherwise well’ neonates with isolated cCMV-related hearing loss within the NHSP.

### Methods

A retrospective database review was completed using the NHS Newborn Hearing Screening Programme (NHSP), and data were collected for all neonates from the North of Tyne region between 1 st September 2013 and 1 st June 2024. Infants who were subsequently diagnosed with a permanent childhood hearing impairment at their audiological assessment were exported and data crossmatched with virology records and clinical databases held by Paediatric Infectious Diseases during the same time period to determine cCMV screening status.

Patient demographics, hearing loss characteristics, and diagnostic outcomes were collected for all infants with a permanent childhood hearing impairment. Neonates with hearing loss were referred for urine CMV PCR testing, with positive cases receiving valganciclovir treatment for 6 months as recommended based on clinical consensus [9], and more recently by European Guidelines [7].

‘Otherwise well’ neonates were defined as those demonstrating no evidence of symptomatic disease at birth (including petechiae, jaundice with conjugated hyperbilirubinemia, hepatosplenomegaly, thrombocytopenia, chorioretinitis, seizures, microcephaly, and intracranial calcifications) [4].

No funding was received for the conduct of this study. As this was a service evaluation the need for formal ethical approval and individual parental informed consent, was waived.

## Results

**Fig. 1:**
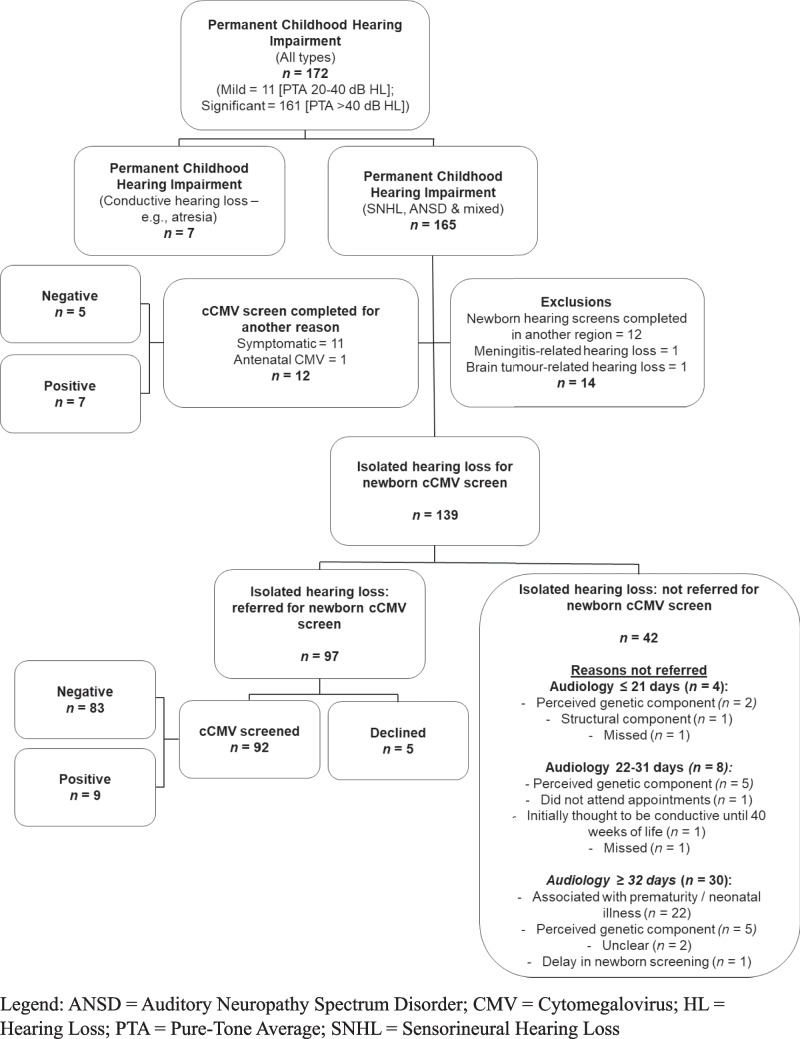
Recruitment and screening

Between 1 st September 2013 and 1 st June 2024 in the North of Tyne region, 57,912 newborn hearing screens were conducted, with 3321 classed as ‘no clear response’ in one or both ears and referred for audiological testing. All infants subsequently diagnosed with a permanent childhood hearing impairment (*n* = 172) were exported from the NHSP database (Fig. 1). Regional birth data was available from 1 st December 2016 to 1 st June 2024 and indicated that 99.5% of newborns completed hearing screens at birth.

Of the 139 ‘otherwise well’ neonates with isolated SNHL, 97 (69.8%) were referred for cCMV screening and 42 (30.2%) were not. Of the 97 referred for CMV PCR, 5 families declined testing due to a strong family history of hearing loss or a known genetic cause. Of the remaining 92, nine (9.8%) were diagnosed with cCMV and 83 (90.2%) were negative. Of the 92 infants tested, 51 (55.4%) were referred for CMV PCR within the recommended 21-day window, and 36 (39.1%) completed testing within this timeframe. All CMV PCR testing was conducted on urine samples.

Among the nine cCMV-positive infants, 2 had bilateral SNHL, and 7 had unilateral SNHL. The hearing levels at diagnostic hearing assessment were derived using Auditory Brainstem Response testing with tone pip stimuli. These ranged from 10 to 50 dB HL (mean PTA = 28 dB HL) in the best ear and from 40 to 100 dB HL (mean PTA = 77 dB HL) in the worst ear. Characteristics of infants screened for cCMV are presented (Table 1).

**Table 1:** Clinical and demographic characteristics of infants with isolated SNHL screened for congenital CMV (cCMV) in the North of Tyne region (2013–2024)

	cCMV positive *n* = 9	cCMV negative *n* = 83
**Gestational age (weeks)**		
≤ 37 weeks	0	16
> 37 weeks	9	67
**Birth weight (g)**	3247 (203.5)	3282.9 (502)
**Sex**		
Male	4	43
Female	5	40
**Ethnicity**		
White	8	63
Asian	0	13
Other	1	3
Unknown	0	4
**Age at audiology (days)**	17 (13–20)	19 (16–22)
**Age at cCMV PCR referral (days)**	21 (19–24)	21 (18–24)
**Age at cCMV PCR result (days)**	22 (20–25)	22 (19–25)
**Age at treatment (days)**	24 (20–25)	-

Age-related variables are presented as median (interquartile range) in days

Birth weight is presented as mean (standard deviation) in grams

The primary reasons why neonates were not referred for cCMV testing (*n* = 42) were prematurity or neonatal illness (*n* = 22, 52.4%) and presumed genetic aetiology (*n* = 12, 28.6%). Twelve of these 42 infants later underwent retrospective dried blood spot (Guthrie card) testing, with one positive result.

## Discussion

Findings from our targeted cCMV pathway evaluation indicate that among 92 neonates with SNHL without apparent aetiology, 1 in 10 had cCMV, a proportion consistent with previous estimates [4]. SNHL is however a heterogeneous condition with multiple potential aetiologies, including genetic mutations, perinatal hypoxia, and other congenital infections (i.e. toxoplasmosis) [10]. While our findings demonstrate the value of cCMV screening in enabling early antiviral treatment, it is important to acknowledge that cCMV infection may be an incidental rather than causal finding in some infants given its significant global seroprevalence [11].

In our cohort, two thirds of ‘otherwise well’ neonates with confirmed SNHL were offered cCMV testing. The majority of infants not tested (52.4%) were those with complex neonatal illnesses requiring protracted neonatal hospital stays, which delayed initial audiology assessments beyond 3 weeks of life. These children are at increased risk of both cCMV and SNHL, and therefore represent an important subgroup for early diagnostic consideration [12].

As early antiviral treatment may be associated with improved hearing outcomes, there is a clear clinical need for timely cCMV screening of hospitalised neonates, preferably before 3 weeks of life [5]. An initial diagnostic audiological assessment is required by audiology standards, which according to NHSP guidelines should be completed for > 90% of referred infants by 44 weeks of gestational age [13]. Locally, we aim to complete audiological assessments by 43 weeks of gestational age or within 3 weeks of referral. Not meeting these targets may delay cCMV screening. The guideline requires the hearing loss to be confirmed; however, our local data evidenced that no permanent childhood hearing impairment diagnoses were subsequently revoked in these neonates, supporting the implementation of our pathway, before confirmation of SNHL is obtained. Although the greatest benefit is associated with earlier treatment initiation, recent European Guidelines propose that treatment may be considered up to 3 months of life [7, 14, 15]. For hospitalised infants, CMV testing can be simply performed on urine obtained during the neonatal stay before 3 weeks of age. Additionally, European Guidelines recommend CMV testing at birth in very preterm (< 32 weeks gestational age) and very low birth weight (< 1500 g) infants, in order to differentiate between congenital and postnatal CMV infection [7].

Nonetheless, practical challenges exist in obtaining CMV samples within 21 days in *‘otherwise well’* neonates who fail their newborn hearing screen but are discharged before audiology follow-up. Without a defined mechanism for early outpatient sample collection, it is likely that some infants may miss the diagnostic window. In such cases, retrospective dried blood spot PCR may be useful, but reduced sensitivity and delayed results limit its immediate clinical utility [16, 17]. In our cohort, all CMV PCR testing was performed on urine samples. A small number of positive cases had testing slightly beyond 21 days of age (up to day 25). While postnatal CMV infection cannot be entirely excluded, the presence of confirmed sensorineural hearing loss and limited exposure to typical postnatal transmission routes makes congenital infection the more clinically plausible explanation. Of the five infants tested after day 21, three had cranial ultrasound findings consistent with cCMV infection, supporting a congenital rather than postnatal origin. Nonetheless, we acknowledge that urine PCR after 21 days cannot definitively distinguish congenital from postnatal infection, and there remains a small risk of misdiagnosis. Future protocols incorporating dried blood spot PCR or maternal serology could improve diagnostic certainty when early testing is missed.

The second most common cause for missed cCMV testing is due to a perceived genetic cause (i.e. family history or syndromic presentation). Considering that genetic causes and cCMV can coexist, and treatment of cCMV can improve hearing outcomes, screening for cCMV even where there may be a genetic element still has value and could be improved with increased awareness and improved counselling.

Finally, an important limitation of targeted screening is that it fails to detect infants with cCMV who have normal hearing at birth but later develop progressive or late-onset hearing loss. Since our pathway is only triggered by failed newborn hearing screens, these infants may remain undiagnosed and miss treatment opportunities.

Despite these limitations, we believe the described approach offers identification and therefore treatment to infants with cCMV SNHL that would otherwise be missed.

## Data Availability

Anonymised data is provided within the manuscript.
